# The Impact of Preventive Strategies Adopted during Large Events on the COVID-19 Pandemic: A Case Study of the Tokyo Olympics to Provide Guidance for Future Large Events

**DOI:** 10.3390/ijerph20032408

**Published:** 2023-01-29

**Authors:** Yina Yao, Pei Wang, Hui Zhang

**Affiliations:** Department of Engineering Physics, Tsinghua University, Beijing 100084, China

**Keywords:** COVID-19, Tokyo Olympics, risk assessment, preventive measures, large event

## Abstract

This study aimed to analyze the impact of hosting large events on the spread of pandemics, taking Tokyo Olympics 2020 as a case study. A risk assessment method for the whole organization process was established, which could be used to evaluate the effectiveness of various risk mitigation measures. Different scenarios for Games participants and Japanese residents during the Tokyo Olympics were designed based on the infection control protocols proposed by the Olympic Committee and local governments. A modified Wells–Riley model considering the influence of social distance, masking and vaccination, and an SIQRV model that introduced the effect of quarantine and vaccination strategies on the pandemic spread were developed in this study. Based on the two models, our predicted results of daily confirmed cases and cumulative cases were obtained and compared with reported data, where good agreement was achieved. The results show that the two core infection control strategies of the bubble scheme and frequent testing scheme curbed the spread of the COVID-19 pandemic during the Tokyo Olympics. Among Games participants, Japanese local staff accounted for more than 60% of the total in positive cases due to their large population and most relaxed travel restrictions. The surge in positive cases was mainly attributed to the high transmission rate of the Delta variant and the low level of immunization in Japan. Based on our simulation results, the risk management flaws for the Tokyo Olympics were identified and improvement measures were investigated. Moreover, a further analysis was carried out on the impact of different preventive measures with respect to minimizing the transmission of new variants with higher transmissibility. Overall, the findings in this study can help policymakers to design scientifically based and practical countermeasures to cope with pandemics during the hosting of large events.

## 1. Introduction

The COVID-19 pandemic has threatened human health, the economy, and society in many countries [1,2,3] since 2019. COVID-19 has brought widespread interference to sporting events, with the majority of them coming to a standstill or being played without spectators [4]. The 2020 Summer Olympic and Paralympic Games in Tokyo were postponed until July 2021 [5]. Large-scale vaccine deployment was expected to signal the end of the pandemic by 2021. However, by 1 July 2021, only 14.6% of the total population in Japan was fully vaccinated [6], which was far lower than the estimated threshold of 70% needed to achieve herd immunity [7]. The emergence of new SARS-CoV-2 viruses made the situation even worse, since the new variants were more transmissible, especially the Delta variant [8]. Due to the exponential rise in confirmed cases, the fourth emergency state was declared in Tokyo only two weeks prior to the Olympics [9,10], which lasted from 12 July to 30 September. Facing these foreseen challenges, the delayed Tokyo Olympics was successfully held from 23 July to 8 August 2021. With only 41 athletes and 822 non-athlete personnel testing positive for COVID during the Tokyo Olympics and Paralympics [11], the Games were reported to be safe for participants and Japanese residents.

The successful hosting of the Tokyo Olympics benefited from the infection control strategies applied at the events, among which the frequent testing scheme and bubble scheme played an important role in curbing the spread of COVID-19 [12,13]. The bubble scheme includes a series of measures to separate participants from the general public. During the first 14 days after entering Japan, foreign personnel were prohibited from using public transportation [14]. With respect to the frequent testing scheme, athletes were required to undergo daily SARS-CoV-2 PCR testing. Other personnel received regular PCR tests at different frequencies depending on their levels of contact with athletes [14]. Extra behavioral rules and preventive measures applied for the Games participants are listed in Table 1 [14]. No spectators were allowed to attend the Olympics except at some remote locations outside Tokyo. COVID-19 vaccination was encouraged but not mandatory for all Games participants [15]. It was found that more than 80% of the participants were fully vaccinated [16], although the vaccination rate for local residents was only 14.6%. For the local residents, some preventive suggestions were proposed, such as refraining from non-essential outings [9].

Considering the Tokyo Olympics as a unique case, this study aimed to assess the risk of mass gathering events by taking the preventive and control measures into account based on the establishment of a risk-based approach. Multiple infection risk models were developed to provide quantitative evaluations of the risk level of infectious disease transmission, such as the susceptible-infected-recovered (SIR) model, the environmental exposure model and the logistic model [17]. The SIR model [18] estimates the temporal evolution of the infection risks in a population, which is useful for assessing preventive measures (e.g., quarantines and vaccination) [19]. To forecast trends in COVID-19 infection, many scholars have extended the SIR model to the time-dependent SIR model, SEIR model, SEIRD model, etc. [20,21,22,23,24]. The time-dependent SIR model with time-varying transmission and recovery rates was developed by Chen et al. [20] to predict the spread of COVID-19. Prasad et al. proposed a QSIR method by introducing a compartment of quarantine based on the SIR model [22], and the optimal control method was applied to estimate the parameters. Singh et al. [23] presented a generalized SIR (GSIR) model, which is a comprehensive model that incorporates multiple waves of daily positive cases.

While SIR-type models are suitable for simulating large-scale behaviors of a pandemic with respect to time, the environmental exposure model can evaluate the infection risks from individual pathways through the integration of pertinent source–environment–receptor pathways [25]. Among the environmental exposure models, the Wells–Riley model is commonly used to assess the risk of SARS-CoV-2 infection [26,27], where the quantum is identified as the minimum dose of airborne virus to cause infection [28]. The infection risk in the Wells–Riley model depends on the exposure time, ventilation, and initial proportion of infected individuals [29,30,31]. Xu et al. [30] evaluated the impact of preventive measures, such as increased ventilation and air filtration, on reducing the infection risk of COVID-19 in U.S. schools. Sun et al. [32] developed a modified Wells–Riley model by introducing two critical indices—social distancing probability and ventilation effectiveness. Their model can help to quantitatively evaluate the impacts of social distancing and ventilation on curbing virus transmission. Hence, appropriate prediction models should be selected and modified to accommodate the different scenarios involved in the Tokyo Olympics.

The aim of this study was to establish a feasible risk assessment method to evaluate the effectiveness of preventive measures and use it to provide suggestions for the government to host a safe large event under the pandemic crisis. First, based on actual conditions at the Tokyo Olympic Games, various scenarios and models were developed according to a series of risk mitigation measures proposed by the International Olympic Committee (IOC) and Japanese government. The proposed Wells–Riley model and SIQRV model were used to predict the transmission of COVID-19 during the Tokyo Olympics. By comparing the predicted results with the actual data, the ability of our risk assessment method to evaluate the effectiveness of prevention measures on the pandemic was verified. Second, according to the simulated results, the risk management flaws of the Tokyo Olympics were identified, and several improvement countermeasures were proposed, such as applying different behavior rules for Japanese staff, increasing the frequency of testing for media personnel, and enhancing quarantine and immunization levels for residents. Lastly, we used the risk assessment method to evaluate the effectiveness of various control measures that may be implemented during a future large event, and thus suggested scientific and effective strategies for hosts to safely organize future large events.

## 2. Methods

The level of risk to the city from existing hazards and threats can be affected by a large event; conversely, the city’s current level of risk can influence the level of risk from event activities, either positively or negatively. In this study, the risk interdependency profile presented by the pandemic between the host city of Tokyo and the event, namely the Olympic Games, was explored. The risk profile of the event refers to infection risk inside the bubble among Olympic-related personnel, and the risk profile of the host city refers to the infection risk outside the bubble among the Japanese residents in Tokyo. The risk involved in the bubble scheme needs quantitative assessments to evaluate the effectiveness of risk mitigation measures adopted during the event. The case design for the bubble scheme and the models used for the risk evaluation are introduced in detail in the following subsections.

### 2.1. Base Case Design for the Bubble Scheme

To account for the activities and locations, we classified the individuals in Tokyo into two categories, namely Olympic-related personnel (participants in the Tokyo Olympic Games) and Japanese local residents. Olympic-related personnel included athletes, Olympic officials, media personnel and Japanese local staff (including volunteers, service staff, etc.). On the basis of the reported data, the total number of athletes participating in the Tokyo Olympic Games was 11,656, coming from about 200 countries around the world. The number of foreign Olympic officials and media totaled about 16,000. Given that the exact number of local staff was not available, it is assumed to be 30,000 in this study. Depending on the bubble scheme, two categories of areas were classified, including the “inside the bubble” areas and “outside the bubble” areas. The “inside the bubble” areas included the official accommodations, competition venues and Olympics Village, whereas shopping and visitor areas and the citywide region belonged “outside the bubble”. Two kinds of transport were involved: public transport (subway services) and dedicated transport (specialized buses).

**Figure 1:**
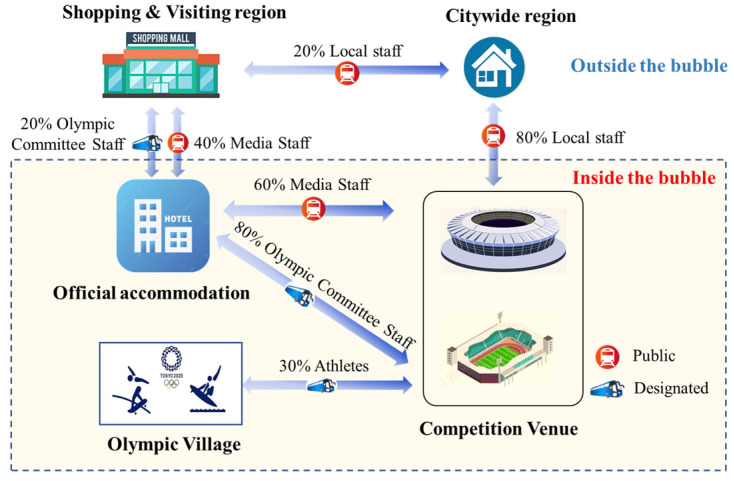
The routes and activities of different groups of people during the simulation period.

Based on the prevention and control protocols implemented by the IOC and Japanese government, various scenarios for individuals in different groups were designed. Their activities, routes and transportation are illustrated in Figure 1. For the simulation involving Olympic-related personnel, the simulation time was the period of the Tokyo Olympic Games, lasting 16 days (from 23 July to 8 August). In order to explore the infection risk brought to the society by the Games, the overall infection trend for Japanese residents in the citywide region of Tokyo was simulated for 14 weeks (from 28 June 2021 to 3 October 2021). In addition, several assumptions were made in the scenario design: (1) Athletes were not allowed to travel outside the Olympic Village and competition venues; (2) in addition to locations inside the bubble, Olympic officials could also travel to shopping and visitor areas by taking designated transportation; (3) media personnel had to comply with the 14-day quarantine restrictions at the beginning of the simulation. As a result, they were permitted to travel using public transportation; (4) Japanese local staff could freely enter and exit the bubble through public transportation; (5) Japanese local residents not associated with the Games were not allowed to enter or exit the bubble.

**Figure 2:**
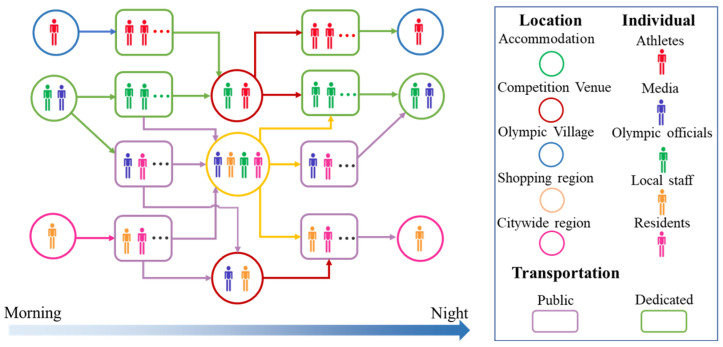
Persons identified in different groups (indicated by different colors) come into contact at different locations and through different transportation modes (indicated by different colors and shapes) during a day.

In accordance with the behavioral rules presented above, the schedule and routes for different groups of Games-concerned personnel are further illustrated in Figure 2. Each symbol represents a specific group of persons, location or type of transportation. The risk of infection during a person’s stay in different locations and using different modes of transportation over a day can be estimated by predictive models. Considering the differences in each scenario, several distinct risk evaluation methods were adopted to assess the risk inside and outside the bubble, as listed in Table 2. Owing to the suitability of SIR-type models for simulating large-scale behaviors of a pandemic with regard to time, the SI model was chosen to predict the daily COVID-19 transmission rate in the competition venues and shopping and visitor areas. Given that infected Games-associated personnel were tested frequently and isolated immediately once they tested positive, we used the SI model, which did not include a compartment for recovered patients. For the confined indoor environments of transportation modes, the Wells–Riley model was applicable to evaluate the infection risk among passengers by taking into account factors such as ventilation, exposure duration, social distancing and the protective effect of masks. Additionally, in order to assess the societal level of risk related to hosting the event, a reliable SIQRV model was constructed to estimate the overall infection trend for residents in the citywide region of Tokyo. The impacts of the infection control strategies of quarantine and vaccination were introduced into the SIQRV model. It should be noted that the spread of disease at official accommodations and the Olympic Village were not considered, since these locations were places where people stayed at night.

### 2.2. Prediction Model Used Inside the Bubble

For the activities of Games-associated personnel in the base case, the number of people moving from *n*-th location to *m*-th location is defined as $W_{nm}$. The numbers of passengers in different states (susceptible or infected) moving from the *n*-th location to the *m*-th location are quantified as,

(1) $$S_{nm}=S_{n}W_{nm}/N_{n}$$

(2) $$I_{nm}=I_{n}W_{nm}/N_{n}$$

where $S_{n}$, $I_{n}$, $N_{n}$ are the number of susceptible people, infected people and all people who are involved in the *n*-th location, respectively. The SI model is governed by Equation (3), which is used for the prediction of epidemiological dynamics of COVID-19 at different locations

(3) $$\frac{dS}{dt}=-\beta IS/N,\;\frac{dI}{dt}=\beta IS/N$$

where *S* and *I* are the number of susceptible people and infected people, respectively; *β* is the transmission rate; *N* is the total number of occupants; *t* is the time. Then, the analytic solution of the SI model can be obtained as:

(4) $$I=\frac{N}{\left(1+\left(\frac{N}{I_{0}}-1\right)*\exp\left(-\beta t\right)\right)}$$

where $I_{0}$ is the number of initial infectors. The transmission rate was estimated according to a real case of COVID-19 clustered infection that occurred on the “Z22” train [33]. At least 35 passengers aboard a train that set out from Lhasa in the Tibet autonomous region on 15 August 2022 and arrived in Beijing on 17 August 2022 tested positive for COVID-19 [33]. The train, Z22, had 10 stops during its over 40 h journey. Then, the transmission rate *β* was estimated as 0.089 per hour, and this value was used in the SI model in our study.

The Wells–Riley model was adopted to predict the transmission trend of the virus in the public and designated transportation cabins. The basic Wells–Riley equation is presented as below:

(5) $$P=1-\exp\left(-\frac{I_{0}qpt}{Q_{m}/N}\right)$$

where *P* is the probability of infection; $I_{0}$ is the number of initial infectors; *p* is the pulmonary ventilation rate of susceptible people (m^3^/h); *t* is the exposure time (h); $Q_{m}$ is the fresh air volume per person (m^3^/h∙per); q is the quanta generation rate (h^−1^). Sun et al. [32] introduced a social distance index $D_{s}$ into the Wells–Riley to study the effect of social distancing, which represents the correlation between the statistical probability of droplets containing virus and the distance they travel.

(6) $$D_{s}=(-18.19ln\left(d\right)+43.276)/100$$

where $D_{s}$ is the social distance index, and *d* represents the social distance (m). Taking the filtering effect of masks into account, a mask index $E_{M}$ is defined, representing the effectiveness of masks in mitigating the risk of infection among susceptible people. Wearing a mask can diminish both the amount of virus exhaled by an infected person and the amount of virus inhaled by a susceptible person. The mask index is expressed as:

(7) $$E_{M}=\left(1-\;f_{I}\eta_{I}\right)\left(1-\;f_{S}\eta_{S}\right)$$

where $E_{M}$ is the mask index, and $\eta_{I}$, $\eta_{S}$ are the filtration efficiency for exhalation and respiration, respectively. $f_{I}$, $f_{S}$ are the proportion of infector and susceptible individuals wearing masks. On the basis of previous studies, both filtration efficiencies of the masks were assumed to be 50% [34]. To further consider the protective effect of vaccination, we extended the Wells–Riley model by introducing the vaccination index v, which represents the influence of vaccination on the infection risk reduction. The vaccination index can be represented as:

(8) $$v=\left(1-\;f_{v}v_{e}\right)$$

where v is the vaccination index, $v_{e}$ is the effectiveness of the vaccination, and $f_{v}$ is the proportion of individuals who are vaccinated. Based on the vaccination data in Japan as of July 2021, the values of $f_{v}$ for the Japanese residents and Olympic-related personnel were assumed to be 0.25 and 0.85, respectively. The protection efficiency of vaccination was set as 0.7 in this study.

The Wells–Riley model was modified as Equation (9) through the integration of social distance, mask and vaccination indices:

(9) $$P=1-\exp\left(D_{s}E_{M}v\frac{qptI_{0}}{Q_{m}/N}\right)$$

The infection probability *p* presented as Equation (9) was calculated for the indoor scenarios. As the quanta emission rate q is an unknown quantity that depends on many factors, a wide range (14 h^−1^ to 1190 h^−1^) for the SARS-CoV-2 virus was suggested or derived from data reported in the previous literature [35,36,37]. Similarly, the quanta generation rate for COVID-19 used in this study was estimated from the case of the “Z22” train. The social distance and fresh air volume per person were estimated based on the designed regulations (Table 3). The proportion of individuals wearing masks and the vaccination rate for the population on the train were set as 40% and 60%, respectively. Consequently, the quanta generation rate was calculated to be 452 h^−1^.

### 2.3. Prediction Method Used for Citywide Region

**Figure 3:**
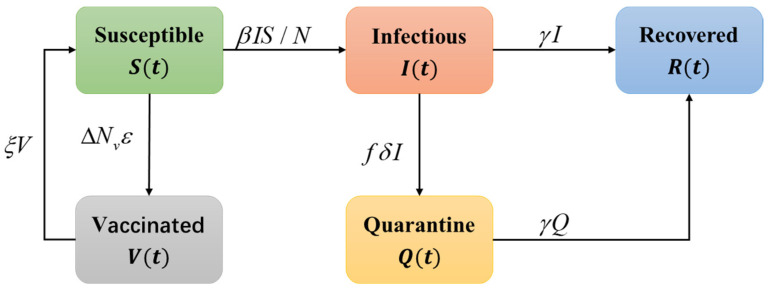
Generalized flow chart of SIQRV compartment model.

Due to the hosting of the Tokyo Olympic Games, some intervention strategies were implemented by the Japanese government for the residents outside the bubble, such as quarantine, vaccination and travel restrictions. To analyze the epidemic dynamics of COVID-19 among residents in Tokyo, we constructed a SIQRV model based on the SIR model by considering the impact of preventive measures. In the SIQRV model, two new compartments were added to the SIR model. The generalized flow chart of the SIQRV compartment model is presented in Figure 3. Q is the quarantine compartment, representing individuals who are quarantined. V is the vaccination compartment, representing the fully vaccinated people. We assume that the population acquired immunity immediately after vaccination. Differential equations of the SIQRV model are as follows:

(10) $$\frac{dS}{dt}=-\beta IS/N-\Delta N_{v}v_{e}+\xi V$$

(11) $$\frac{dI}{dt}=\beta IS/N-\gamma I-\mu I$$

(12) $$\frac{dR}{dt}=\gamma I+\gamma Q$$

(13) $$\frac{dQ}{dt}=\mu I-\gamma Q$$

(14) $$\frac{dV}{dt}=\Delta N_{v}v_{e}-\xi V$$

where S, I, Q, R, and V represent the numbers of susceptible, infected, quarantined, removed and vaccinated population, respectively. γ is the removal rate (1/γ represents the average infectious period), μ is the quarantine rate, $\Delta N_{v}$ is the number of newly vaccinated people per day, $v_{e}$ is the effectiveness of the vaccination, and ξ is the period of immunity. The total population *N* is considered to remain constant:

(15) S+I+Q+R+V=N

The basic reproduction number $R_{0}$ of the SIQRV model was calculated according to the next-generation matrix method [38] for compartmental models. Setting F to be the emergence rate of newly infected people in the compartments, let $V^{+}$ be the transfer rate of individuals entering the compartment through all other routes, and $V^{-}$ be the transfer rate of individuals out of compartment, $V=V^{-}-V^{+}$. The matrix of F and V for all of the compartments in our model is obtained as:

(16) $$F=\left(\begin{array}{c} \beta IS/N-\mu I\\ \mu I\\ 0\\ 0\\ 0 \end{array}\right),\;V=\left(\begin{array}{c} \gamma I\\ \gamma Q\\ \beta IS/N+\Delta N_{v}v_{e}-\xi V\\ \gamma I+\gamma Q\\ -\Delta N_{v}v_{e}+\xi V \end{array}\right)$$

Define $DF(x_{0})=\left(\begin{array}{ll} Y & 0\\ 0 & 0 \end{array}\right)$, $DV(x_{0})=\left(\begin{array}{ll} Z & 0\\ J_{3} & J_{4} \end{array}\right)$ where Y and Z are the m × m matrices defined by:

(17) $$Y=\left[\frac{\partial F_{i}}{\partial x_{j}}(x_{0})\right],\;Z=\left[\frac{\partial V_{i}}{\partial x_{j}}(x_{0})\right],\;1\leq i,\;j\leq m.$$

Let the right-hand side of Equations (10)–(14) equal zero; the disease-free equilibrium point $x_{0}=\left(S_{0},\;0,\;0,\;0,\;V_{0}\right)$ is obtained, where $S_{0}$, $V_{0}$ are the number of susceptible and vaccinated people at the disease-free equilibrium point $x_{0}$. They satisfy the conditions of $S_{0}+V_{0}=N$ and $V_{0}=\Delta N_{v}v_{e}/\xi$. Then, the value of $S_{0}$ can also be calculated. Applying the Fréchet derivatives to F and V at $x_{0}$, we obtain

(18) $$Y=\left(\begin{array}{ll} \frac{\beta S_{0}}{N}-\mu & 0\\ \mu & 0 \end{array}\right),\;Z=\left(\begin{array}{ll} \gamma & 0\\ 0 & \gamma \end{array}\right)$$

then,

(19) $$R_{0}=\rho \left(FV^{-1}\right)=\frac{\beta S_{0}-\mu N}{\gamma N}$$

where ρ(⋅) is the spectral radius of a matrix, $R_{0}$ represents the expected number of new infections generated in the total population by one typical infector before his/her recovery [39]. If $R_{0}>1$, the number of infections is likely to increase and there will be an outbreak, and if $R_{0}<1$, the number of infections is likely to decline and there will be no outbreak. Since $S_{0}$ is related to the parameter of $V_{0}$, the $R_{0}$ is not a constant but a variable that depends on the initial fully vaccinated proportion of the population $V_{\mathrm{initial}}$.

The proportion of Japanese residents who were newly vaccinated each day relative to the population was obtained from the website “Our World in Data. Coronavirus (COVID-19) Vaccinations” [6]. Setting the search interval as the simulation period from 28 June 2021 to 3 October 2021, we acquired the proportion of fully vaccinated people and daily COVID-19 vaccine doses administered in Japan. The proportion of fully vaccinated people in Japan at the beginning of the simulation period (on 28 June 2021) was obtained as 11%. The average number of daily vaccine doses administered during the simulation period was calculated to be 1.12%. Since people were fully vaccinated only after two doses of vaccines were administered, it is assumed that about 0.56% of the total population were newly vaccinated each day during the simulation period.

## 3. Results

### 3.1. Base Case

#### 3.1.1. Prediction of COVID-19 Cases among Olympic-Related Personnel in the Bubble System

Based on the reported data, the number of daily COVID-19 positive cases during the Olympics was extracted from the OG website [11]. A total of 436 Olympic-related personnel tested positive from 1 July to 8 August. Among the 436 positive cases, 87 were identified as pre-Olympics (1 July to 22) and 349 as during-Olympics (23 July to 8 August) cases [11]. However, 82,246 Japanese residents in Tokyo were confirmed positive during the same period, equal to 0.59% of the total population in Tokyo. Given that the infection rates associated with the Olympics were much lower than that in Tokyo, the implementation of preventive and control measures was considered to be relatively successful in reducing infection risk inside the bubble.

**Figure 4:**
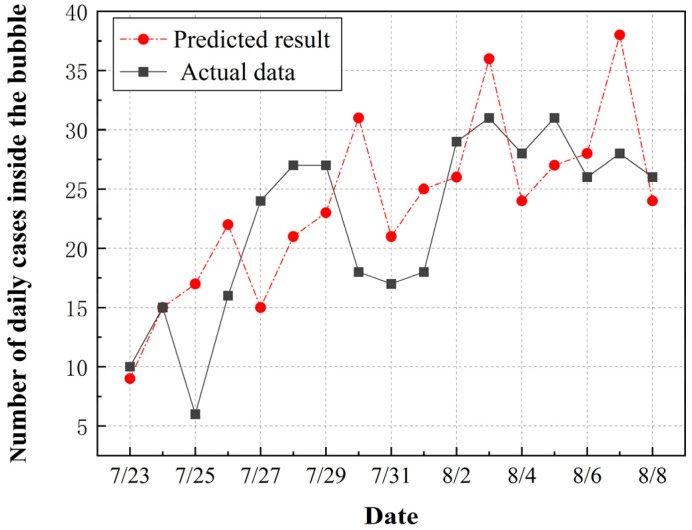
Comparison between predicted results and actual data for daily new cases among Olympic-related personnel inside the bubble.

Based on the risk assessment methods introduced in Section 2, the numbers of positive cases for different groups of Games-associated personnel inside the bubble were predicted. The parameters used in the simulation are listed in Table 4. Algorithm 1 presents the detailed simulation procedure in the form of pseudocode. Figure 4 presents the comparison of predicted results and actual data for daily confirmed cases. The predicted temporal trend was generally consistent with that of the actual data. The cumulative number of confirmed cases among Games-associated personnel during the studied period was calculated to be 399, which varied 14.3% from the actual number of infections. It reveals that the design of the base case and the prediction model used in this study were applicable to the transmission of COVID-19 inside the bubble. In terms of the simulation results, the number of daily positive cases decreased sharply after a four consecutive days of increases. This variation was due to the different testing frequencies among Games-concerned personnel. The frequent COVID-19 testing scheme was found to be an effective countermeasure to contain the spread of the pandemic.
**Algorithm 1.** Simulation procedure for the Olympic-related personnel1: Input model parameters: $S_{0}$, $I_{0}$, β, $f_{t}$, δ for each scene 2: Initialization: *t* = 0 (Day) begin of the simulation time 3: **repeat** 4:   Calculate the number of newly infected cases in each scene using the corresponding model. 5:   Obtain the number of cumulative cases among each type of Olympic-related personnel after one day. 6:   Calculate the remaining undetected infected cases based on the parameter of nucleic acid test frequency $f_{t}$ and detection accuracy δ 7:   Update the number of $I_{0}$, $S_{0}$ for each scene 8:   *t* = *t* + 1 9: **until**
*t* = 16, the end of the simulation time **Output**: The number of newly infected cases for each day and the number of cumulative cases for the whole simulation time.

**Figure 5:**
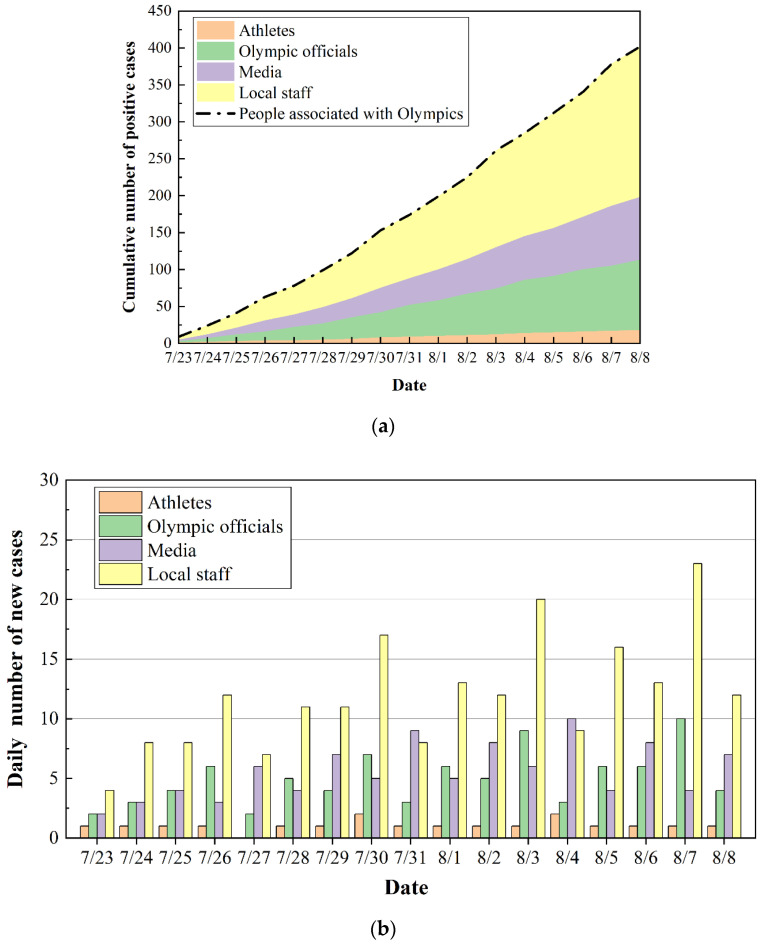
Predicted results: (**a**) variation in cumulative cases; (**b**) daily numbers of new cases for people in different roles.

Figure 5 shows the predicted cumulative number and daily number of positive cases among each population with different roles. One can see that the cumulative number of cases presented a linear growth trend rather than exponential growth (Figure 5a), which was attributed to the implementation of specific risk mitigation measures. The total numbers of infected athletes, Olympic officials, media and local staff were predicted to be 18, 83, 96 and 202, respectively. Due to the most stringent travel restrictions and the most frequent testing requirements, only 18 athletes were estimated to have been infected, which was close to the reported and confirmed number of 21 cases [12]. As illustrated in Figure 5b, the daily number of cases for athletes was calculated as 0, 1 or 2. This may deviate from the actual situation due to the simplification of our model, which could not forecast clusters of infections among athletes. With regard to the numbers of cumulative and daily confirmed cases, it can be found that Japanese local staff accounted for more than 60% of positive cases. This resulted from the largest population of Japanese local staff and the longest contact times between them and other populations. Additionally, although the Olympic officials were tested (once every 2 days) more frequently than media personnel (once every 4 days) in the base case, our results indicate that more Olympic officials were infected compared with media personnel. This is because Olympic officials always traveled together by taking designated transportation, which was likely to result in cluster infections. On the other hand, media personnel mainly traveled by public transportation alone or in small groups, yielding a relatively lower infection risk.

**Figure 6:**
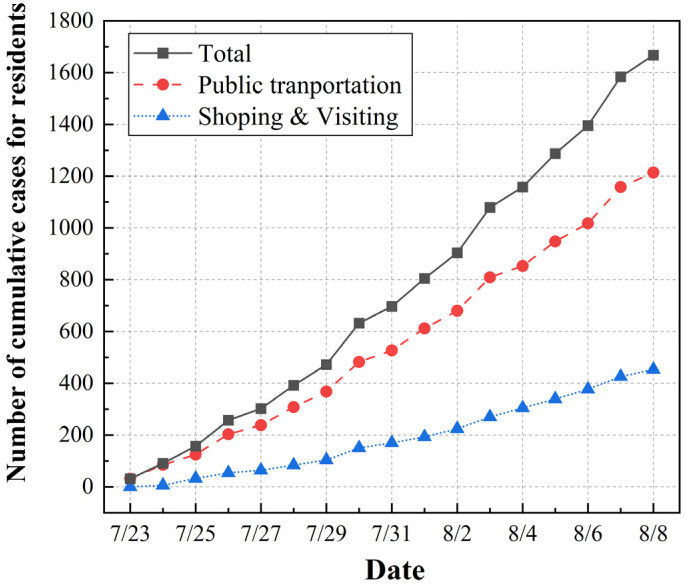
The predicted cumulative number of positive cases for residents infected by Olympic-related personnel.

In the base case, the virus may have been brought out of the bubble, given that the activities of shopping, visiting and using public transit were allowed for a some of the Olympic-related personnel. The number of residents infected by the Olympic-related personnel was predicted to reflect the infection risk leaking out of the bubble, as shown in Figure 6. The average vaccination rate for the population was assumed to be 25% for the residents during the Olympics. Due to the continuous flows of people in the shopping and visiting scenarios, it was assumed that the transmission rate β was twice that in the case of the “Z22” train. The total number of infected residents was estimated to be 1667, with 1214 positive cases generated in the public transportation scenario and 453 cases in the shopping and visiting scenario. Therefore, the average number of daily positive cases for residents could be calculated as about 104, which represented a very small percentage compared to the reported daily number of positive cases (an average of 3804 confirmed cases were reported per day in Tokyo during the Olympics [24]). Our results provide evidence that the hosting of the Olympic Games had no direct effect on local transmission of COVID-19 among residents. In addition, it can be noticed that the risk of infection was greater in the scenario of public transportation than in the scenario of shopping and visiting. Given that the range of activities was not limited among the local staff, a large proportion of infected residents were infected by local staff on public transport.

#### 3.1.2. Prediction of COVID-19 Cases among Japanese Residents in the Citywide Region

In contrast to the quite low proportion of positive cases in the bubble system, the spread of COVID-19 among the citywide population of Tokyo was completely the opposite. On 1 July 2021, there were approximately 673 new cases of COVID-19 in Tokyo; nevertheless, a record increase in cases was reported during the Olympics. The city witnessed a surge in daily COVID-19 cases and continued to struggle with an upward trend in the 2 weeks after the Games. By 13 August, the daily number of confirmed cases in Tokyo reached an alarming peak of more than 5773. As of 28 August, the number of daily cases started to decrease, with only 82 reported cases by 12 October 2021 [11].

As stated in Section 2.3, the SIQRV model was constructed in this study to predict the citywide spread of the COVID-19 pandemic in Tokyo under the impacts of preventive strategies for quarantine and vaccination. The study period was from 28 June to 3 October 2021 (98 days in total) and covered the period of the Olympics. Based on the method introduced in Section 2.3, the proportion of fully vaccinated people $V_{\mathrm{initial}}$ on 28 June 2021 was set as 11%. The proportion of people who were newly vaccinated each day relative to the population was 0.56%. Thus, the value of parameter $\Delta N_{v}$ (defined as the number of newly vaccinated people per day) could be obtained as 78,176 for the citywide region of Tokyo. The initial numbers of susceptible, infected, and vaccinated individuals used for the simulation were obtained from the reported data.

Since the percentage of confirmed cases infected by the Delta variant (i.e., variant B.1.617.2) increased from 21.5% (28 June to 4 July) to 94.0% (23 August to 29 August) [12], it was considered as the main variant transmitted during the Olympics in this study. The estimated basic reproduction number $R_{0}$ of new infections with the Delta variant ranged between 6 and 7, which indicated that it was three times more infectious than the original COVID-19 strain [40,41]. The transmission rate of the Delta variant could be estimated to be 0.6 to 0.7. The parameter settings for the base case are displayed in Table 5. Generally, the quarantine strategy was always adjusted once every 2 weeks in the actual situation. Thus, the value of quarantine rate μ in the SIQRV model was varied every 2 weeks to match the predicted results with actual data. Then, the most appropriate value of μ could be obtained for different times.

**Figure 7:**
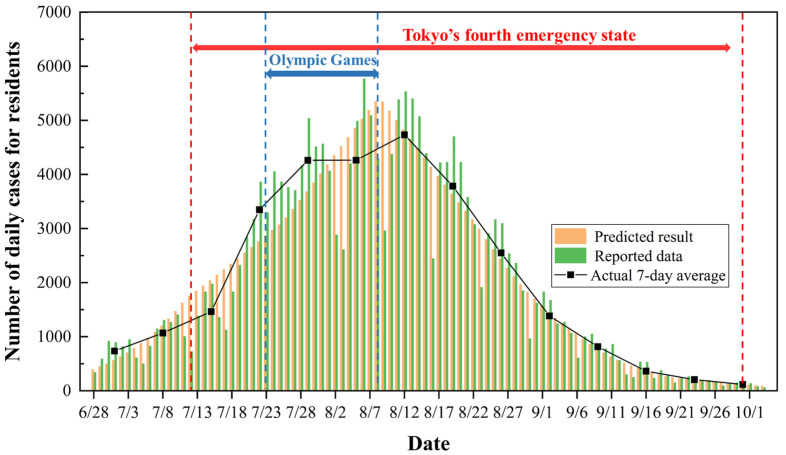
Comparison between predicted results and actual data for daily new cases among Japanese residents in the citywide region of Tokyo.

Figure 7 depicts the comparison between the predicted results and the reported data for daily confirmed cases. As there was always a delay between the timing of case detections and timing of the underlying infections, the number of confirmed cases reported 7 days later was regarded as the number of infected cases on that day. Moreover, the 7-day average number of reported positive cases was also plotted in the figure to better reflect the overall trend in COVID-19 cases. One can see that the trend for the predicted results was generally consistent with that for the actual data, especially in the period from 12 August to 1 October. The difference between the predicted and actual results was identified quantitively. Since the number of daily reported positive cases may depend on some objective factor, such as the number of people receiving a COVID-19 test, the accuracy of the test, etc., we calculated the difference between the predicted and actual results for the cumulative number of weekly positive cases. The mean relative error in the cumulative number of positive cases per week between the two results was calculated to be 10.7%. This difference was considered to be acceptable due to some assumptions and simplifications made in the SIQRV model. Additionally, the predicted total number of cumulative cases during the simulation period was 203,543, which agreed well with the actual results. The good agreement suggests that the surge in positive cases in Tokyo mainly resulted from the high $R_{0}$ of the Delta strain, while the hosting of the Tokyo Olympics was not responsible for that.

To calculate the critical threshold of the quarantine rate $\mu_{c}$ from Equation (14), let $R_{0}<1$, which yields

(20) $$\mu \geq \frac{\beta S_{0}-\gamma N}{N}=\mu_{c}$$

**Figure 8:**
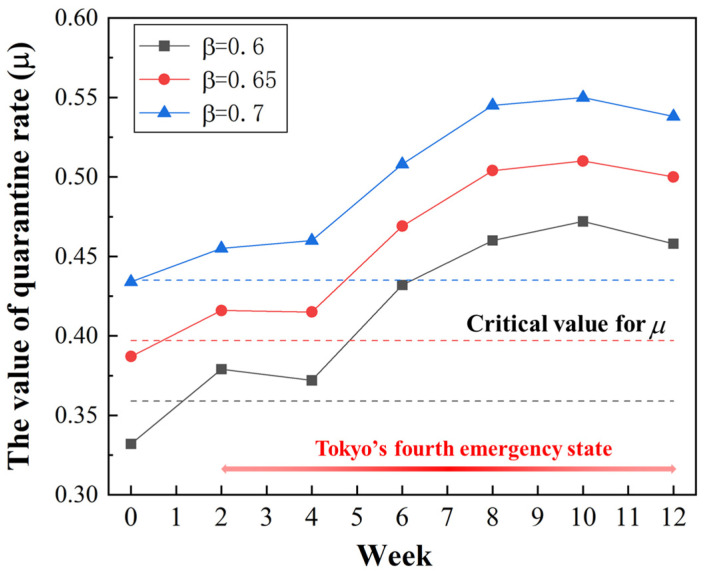
Appropriate quarantine rates per week under different transmission rates.

We obtained $\mu_{c}$ = 0.36, 0.4, and 0.44 for different transmission rates of β = 0.6, 0.65, and 0.7, respectively. The variation in quarantine rate μ with time under different transmission rates was explored, as demonstrated in Figure 8. Only the quarantine rate for the first 2 weeks was lower than the critical threshold (shown by the dotted line). Since Tokyo entered its fourth emergency state from the third week, the value of the quarantine rate increased correspondingly due to the stricter quarantine strategies. It can be observed that the value of the quarantine rate generally increased over time. This was attributed to the variation in the strength of the quarantine strategy, which was adjusted dynamically according to the corresponding pandemic situation. Under the assumption that the detection accuracy was equal to δ=0.8, the frequency of COVID-19 tests for residents ($f_{t}=\mu /\delta$) could be estimated as one test every 1.5–3 days. Our prediction model proved to be useful for analyzing the temporal trend in the stringency of preventive measures (i.e., detection, quarantine).

### 3.2. Analysis of Improved Measures for Tokyo Olympics

The different scenarios involved in the Tokyo Olympic Games and intervention measures adopted during the Games were studied in the base case presented in Section 3.1. Further analysis was carried out to demonstrate the extent of achievable infection risk reduction levels by implementing improved preventive measures. The improved measures consisted of two categories, one for Olympic-related personnel and the other for residents.

#### 3.2.1. Control Measures for Olympic-Related Personnel

As demonstrated in Section 3, only 0.46% of all Olympic-related personnel [12] tested positive during the Games. The containment of COVID-19 spread among Olympics-related personnel was associated with accurate organization measures for the Games. The results here show that frequent COVID-19 testing played a vital role in curbing the spread of COVID-19 and that the behavioral rules involved in the bubble system also helped reduce the infection risk. However, there were still some risk management flaws that posed an increased risk for Olympics-related personnel: (1) Local staff could freely enter and exit the bubble using public transportation; (2) media personnel were allowed to use public transportation and participate in activities not associated with the Games; (3) the frequency of COVID-19 testing for media personnel was relatively low. To address the above management flaws, two improved infection control measures were proposed and investigated. (1) Measure A: Local staff are required to live in official accommodations and take designated transportation. (2) Measure B: The frequency of COVID-19 testing for media personnel is increased from one test every 4 days to one test every 2 days.

**Figure 9:**
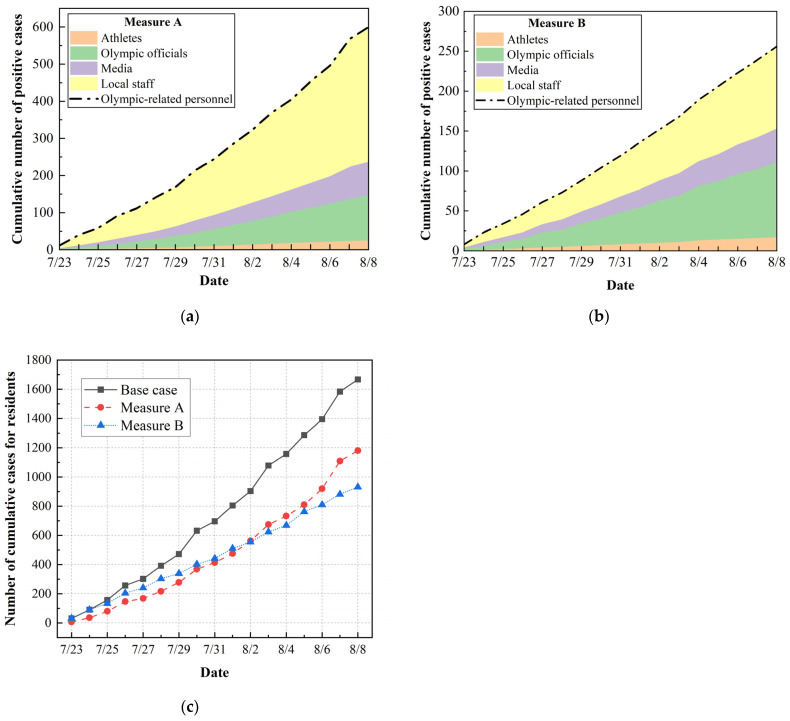
The predicted results under the implementation of Measure A and Measure B: (**a**) The cumulative number of positive cases for Olympic-related personnel under Measure A; (**b**) the cumulative number of positive cases for Olympic-related personnel under Measure B; (**c**) the cumulative number of positive cases for residents under Measures A and B.

The predicted numbers of COVID-19 cases under the implementation of Measure A and Measure B are presented in Figure 9. When applying Measure A, the predicted total number of positive cases for Olympic-related personnel was 600, and the numbers of cases for athletes, Olympic officials, media personnel and local staff were 25, 115, 97, and 363, respectively (Figure 9a). The total number of residents infected by Games-associated personnel was estimated to be 1160 (Figure 9c), a 30% decrease compared with the base case. Overall, although the implementation of Measure A reduced the number of cases among residents to some extent, there were 201 more Olympic-related individuals infected. This is because Measure A diminished the infection risk for residents outside the bubble, while promoting transmission of the virus inside the bubble. Additionally, providing designated transportation and official accommodations imposed an additional financial burden on the host country. Consequently, Measure A was not a good countermeasure to optimize risk management for the Tokyo Olympics considering the balance between safety and the economic aspects.

As illustrated in Figure 9b, when applying Measure B, the total number of positive cases among Olympic-related personnel decreased to 256, and the numbers of cases among athletes, Olympic officials, media personnel and local staff became 17, 94, 42, and 103, respectively. It can be seen that the numbers of cases among both media personnel and local staff were halved. The number of positive cases among residents decreased to 931, representing a reduction of 45% compared to the base case. The simulation results suggest that increasing the frequency of COVID-19 testing for media personnel from one test every 4 days to one test every 2 days contributed to suppressing transmission of the virus within the bubble system. Therefore, it was quite important to ensure frequent COVID-19 testing for all Games-concerned personnel, and Measure B proved to be feasible and effective for improving the risk management of the Games.

#### 3.2.2. Control Measures for Japanese Residents

Although the surge in positive cases was mainly attributed to the high transmission rate of the Delta variant and the low level of immunization among Japanese residents, the increase in the quarantine rate over time also facilitated curbing the spread of COVID-19. Two improved infection control measures are proposed here with respect to quarantine and vaccination. (1) Measure C: The quarantine rate is increased above the critical threshold, ensuring $R_{0}<1$ throughout the study period; (2) Measure D: The initial vaccination rate for the population is increased from 11% to 30% with an interval of 5%. The settings for other parameters are the same as those in the base case (Table 5). 

**Figure 10:**
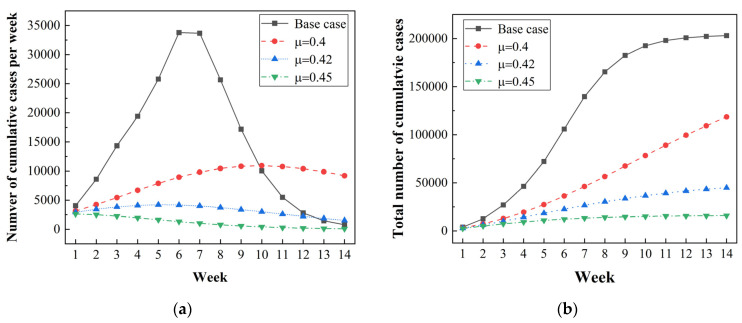

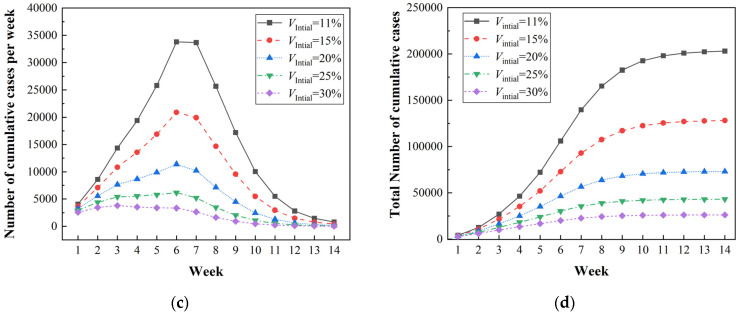
Simulated results under the implementation of Measure C and Measure D, where the transmission rate β=0.6 is used: (**a**) The cumulative number of cases per week under Measure C; (**b**) the total number of cumulative cases under Measure C; (**c**) the cumulative number of cases per week under Measure D; (**d**) the total number of cumulative cases under Measure D.

Figure 10a,b presents the variation in COVID-19 cases under the implementation of Measure C (applying quarantine rate μ≥0.4, larger than the threshold of 0.36). To achieve the expected quarantine rate, the frequency of testing for residents should be maintained with at least one test every 2 days. When applying Measure C, the total numbers of cumulative cases during the studied period decreased sharply, by 41.62%, 77.85% and 92.18%, respectively, for the conditions of μ = 0.4, 0.42, 0.45 compared to the base case (Figure 10b). One can see that the trend in cumulative positive cases per week was different from that of the base case (Figure 10a). This is because the quarantine rate in the base case was varied ever 2 weeks in the range of 0.332–0.472, while it remained constant when Measure C was applied. For the conditions of μ > 0.4, the number of cumulative cases per week decreased after experiencing small growth, which could prevent a sudden surge in the number of infected cases. Compared with the base case, applying Measure C could help relieve pressure on healthcare systems and enhance their emergency response and resilience to a great extent [43]. In general, the implementation of Measure C, i.e., with more stringent testing and a quarantine strategy, would be favorable to avoid mass infections within a short period.

Figure 10c,d shows the predicted results under the application of Measure D, with different initial vaccination rates of 15%, 20%, 25%, and 30%. The simulation results for the base case (11% initial vaccination rate) are depicted for comparison. With higher initial vaccination rates, the number of cases decreased to a large extent, indicating that a more-vaccinated population could control the pandemic transmission. Further, under the assumption that society and hospitals accepted <500 newly infected persons per day during the Games, we propose a cumulative number of cases per week of <3500 as a benchmark for the suppression of infection. To achieve this goal, the initial vaccination rate should be increased to at least 30%, with the number of cases per week lower than 5000 and the total number of cases smaller than 30,000. The results reveal that a high immunization rate prior to the event is essential for the containment of COVID-19. Therefore, it is imperative to enhance the initial vaccination rate among the population before hosting a large event during a pandemic crisis.

### 3.3. Analysis of Preventive Measures for Future Large Events

Under the conditions of a global pandemic, it is of paramount importance to develop scientifically based and practical risk management measures tailored to different events and scenarios to ensure the safety of all involved in a high-profile public event. Due to the successful implementation of preventive measures during the event, there is no doubt that the hosting of the Tokyo Olympics was exemplary for future large events. However, the appearance of new strains and breakthrough infections among fully immunized or vaccinated people will pose challenges for future large events. For example, a new variant Omicron (variant B.1.1.529) was first discovered in November 2021. With more than 50 mutations, Omicron has the ability to increase transmissibility, develop resistance to therapeutic agents, and partially evade existing herd immunity [16]. Previous studies suggested that the basic reproduction number of the Omicron variant ranges from 5.5 to 24, with an average number of 9.5 [44], which is higher than that of the Delta variant and the original SARS-CoV-2 virus. The effectiveness of different infection control measures on the transmission of Omicron was explored using the methods proposed in this study, thus providing detailed risk management recommendations for event organizers and local governments.

**Figure 11:**
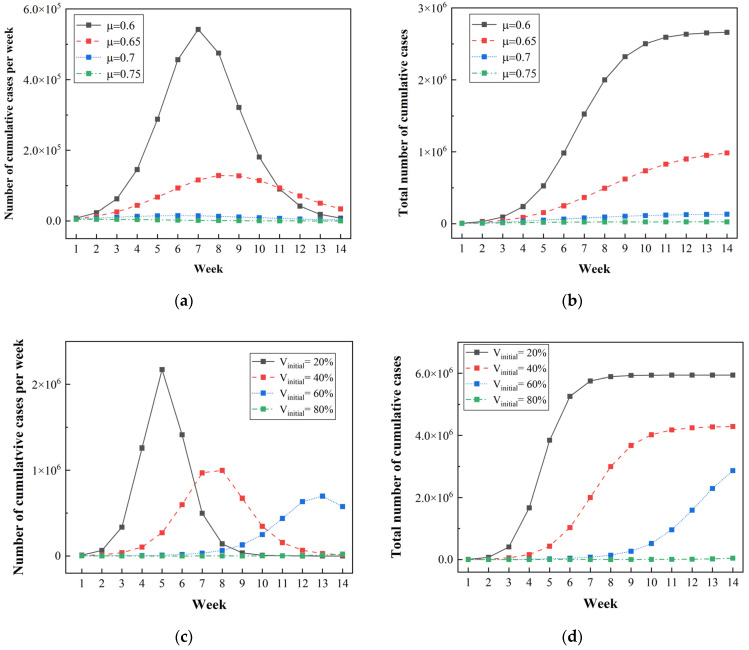
Simulated results under the implementation of different quarantine and vaccination measures during the spread of the Omicron variant: (**a**) The cumulative number of cases per week under different quarantine measures; (**b**) the total number of cases under different quarantine measures; (**c**) the cumulative number of cases per week under different vaccination measures; (**d**) the total number of cases under different vaccination measures.

To ensure that the reproduction number $R_{0}$ for Omicron variant is smaller than 1, the critical threshold of the quarantine rate is $\mu_{c}$ = 0.62. Figure 11a,b shows the predicted results for cumulative cases per week and total positive cases at a quarantine rate of 0.6 to 0.75, where the initial vaccination rate remains consistent with the base case. When the quarantine rate equals 0.6, there is an unprecedented spike in positive cases in a short time, with a total of 2,660,000 infections and over 70,000 cases of infection per day. This surge would overwhelm the health care system with thousands of people waiting for isolation and/or treatment. It can be concluded that keeping the quarantine rate below the critical threshold is not feasible for suppression of the pandemic. An acceptable infection rate can be achieved (cumulative number of cases per week <3500 as a benchmark) only when the quarantine rate is equal to 0.75. This means that daily COVID testing needs to be conducted for the whole population.

Figure 11c,d demonstrates the simulation results under the condition of an initial vaccination rate increasing from 20% to 80%, where the quarantine rate is kept at 0.45. When the initial vaccination rate is increased from 20% to 60%, the infection risk still remains at a high level, with a cumulative total of more than 2,000,000 cases. However, when the herd immunity threshold of 80% is reached, the number of positive cases decreases sharply due to the small population of susceptible people. The results suggest that transmission in a pandemic can be reduced to a very low level if an immunization rate of 80% is achieved. In reality, though, the Omicron variant has spread more rapidly than the simulation results due to its combination of increased transmissibility and greater immune escape ability. Consequently, previous strategies adopted during the Tokyo Olympics are not applicable to the transmission of the Omicron variant. Specifically, more stringent quarantine and vaccination strategies should be combined to deal with new variants with higher transmissibility during a large event.

## 4. Discussion

A set of risk assessment methods were developed and used to evaluate the effectiveness of preventive measures, helping to provide suggestions for improving risk management measures for both the Tokyo Olympic Games and future large events. The simulation results indicated that the bubble scheme and frequent COVID-19 testing for Olympic-related personnel both played a vital role, which resulted in a relatively low risk level in the bubble system. However, as the risk level in the citywide region of Tokyo was high, some improvement countermeasures were proposed to mitigate the infection risk. Adopting a more stringent quarantine strategy with a quarantine rate of greater than 0.4 helps in preventing mass infections in a short period of time, thus reducing pressure on healthcare systems. To increases the quarantine rate, more rooms and empty hotels should be prepared as temporary facilities for patients with mild or asymptomatic infection who need to isolate but do not require hospital treatment. Sufficient staff should be trained to provide services for the people isolated in quarantine. In addition, ensuring a high rate of vaccination before the hosting of the Games will help curb the spread of COVID-19 to a great extent.

Although the hosting of Tokyo Olympics was considered to be successful under the public health challenge, banning spectators and international travelers caused massive financial losses for the organizers. It is estimated that the Tokyo Olympics may had suffered a loss of $800 million in ticket sales. In future international sporting events, a suitable balance between safety and economic aspects should be achieved. For instance, allowing fully vaccinated people and international travelers and preserving proper safety guidelines will minimize economic losses incurred by the host country as well as ensure safety. Moreover, since an international sporting event is expected to result in an increased risk of accidents, injuries, and infectious diseases, it is critical for the host government and hospitals to develop and implement a massive casualty preparedness plan to improve their emergency response and resilience. The capacity of hospitals and health institutions should be reinforced given the potential for mass infection. Extra precautionary measures can also be adopted for future large events, such as having a sufficient workforce, providing appropriate training for medical staff, and offering multi-language services to eliminate the language barrier.

While this study provided some quantitative results, it had several limitations. First, the parameters used for the simulation were estimated according to the case of the “Z22” train and previous studies. Actually, the parameters were supposed to vary under different scenarios, making it difficult to obtain their accurate values. This limitation may result in deviations of our simulation results. Second, the areas in the host city were classified into several rough categories. Difference between the competition venues, accommodations and shopping areas were not considered. Third, the scenario design for the population with different roles was simplified to be the same every day. Due to the lack of specific schedules for the Olympic-related personnel, variation in daily travel scenarios was not taken into account. Fourth, since the Delta variant was regarded as the main variant transmitted during the Olympics, the virus parameters for the Delta variant were used in the simulation. Actually, multiple variants were transmitted during the Games, including the Alpha variant, Gamma variant and others. These variants have different viral parameters such as transmissibility, illness severity, and immune evasion, which should be taken into consideration in further research. Fifth, the lack of economic analysis may have influenced the evaluation of the impacts of prevention strategies. Economic losses incurred by the host country due to a public health crisis should be further investigated. Despite these limitations, a quantitative assessment of the infection risk profile in the bubble system under the implementation of preventive measures during the Tokyo Olympics was accomplished. Additionally, improvement measures for the Games were proposed and discussed. These findings can better support strategies to prevent and/or reduce the negative effects of a pandemic crisis on a high-profile public event.

In conclusion, it is necessary to perform more detailed research on the relations between the transmission dynamics of different variants, human behavior, large events, vaccinations, and intervention measures. More data about real cases should be collected to calibrate the parameters used for the simulation. Moreover, the balance between security and economic aspects for host countries needs to be investigated further, especially in light of the impending emergence of new variants.

## 5. Conclusions

In this paper, the impact of a large event on the spread of the COVID-19 pandemic was investigated by treating the Tokyo Olympics as a unique case. A set of risk assessment methods for all stages of the Games was established, including the modified Wells–Riley model and SIQRV model. The method was used to predict the spread of COVID-19 in the bubble system and evaluate the effectiveness of different infection control strategies adopted during the event. The predicted numbers of daily new cases and cumulative cases for Olympic-related personnel and Japanese residents were compared with actual data, yielding good agreement. Our results indicate that local staff, as the largest group with the longest exposure duration, accounted for the largest proportion of total positive cases among Games-concerned personnel. The public transport scenario was more likely to bring the infection risk out of the bubble compared to the shopping scenario. The predicted results for citywide positive cases suggest that the intensity of the quarantine strategy increased over time since Tokyo entered its fourth emergency state. Moreover, the surge in positive cases in Tokyo was mainly attributed to the high transmission rate of the Delta variant and low level of vaccination, while the hosting of the Games was not responsible for that.

Based on our simulation results, risk management flaws during the Tokyo Olympics were identified, and some countermeasures for improvement were proposed. The achievable level of reduced infection risk under the implementation of improved preventive measures was investigated. The results show that increasing the frequency of COVID-19 testing for media personnel from one test every 4 days to one test every 2 days would reduce the number of positive cases among Olympic-related personnel and residents by more than 45%. Offering designated transport and official accommodations is not a good measure for improvement, as it reduces the infection risk for residents outside the bubble while facilitating transmission of the virus inside the bubble. Applying a high quarantine rate of >0.42 and a large initial vaccination rate of 30% would allow achievement of an acceptable level of infection risk (cumulative number of cases per week <3500 as a benchmark), thus preventing a sudden surge and helping to relieve pressure on healthcare systems.

For the safe organization of future high-profile public events, a further analysis was carried out on the impact of different preventive measures on the spread of the COVID-19 pandemic, taking into account the spread of the new Omicron variant. Since the Omicron variant has higher transmissibility and greater immune escaping ability, the previous measures adopted during the Tokyo Olympics are no longer applicable. We propose that more stringent quarantine (with a quarantine rate higher than 0.7) and vaccination strategies (with initial an vaccination rate higher than 80%) should be adopted to curb the transmission of the Omicron variant. Our results can help policymakers to design scientific and effective strategies to host safe, secure and sustainable events under the pandemic crisis. The trade-off between safety and economic activities should be investigated in a future study.

## Figures and Tables

**Table 1:** Infection control measures implemented during Tokyo Summer Olympics in 2021 [14] (International Olympic Committee Tokyo 2020 playbooks).

Intervention Measures	Personnel	Measures Applied
Daily temperature monitoring for 14 days	Athletes, officials, media	Prior to arriving and during stay in Japan
Submission of COVID negative test	Athletes, officials, media	Before arrival
Quarantine for 3 days	Athletes, officials, media	After arrival
Official accommodation	Athletes, officials, media	During stay in Japan
Use of game designated transportation	Athletes, officials,	During stay in Japan
	media	During the first 14 days after entering Japan
Daily antigen COVID test	Athletes	During stay in Japan

**Table 2:** The models used for different scenarios.

Location	Exposure Time per Day	Personnel	Model
Competition Venue	6	Athletes, Olympic officials	SI
Competition Venue	10	Local staff, Media	SI
Shopping and Visitor areas	10	Athletes, Media, Olympic officials, Japanese residents	SI
Public transport	1.5	Media, Japanese residents	Wells–Riley
Dedicated transport	1	Athletes, Olympic officials	Wells–Riley
Citywide region	/	Japanese residents	SIQRV

**Table 3:** Parameters of confined indoor environment used in the Wells-Riley model.

Scenario	Number of Seats	Length (m)	Width (m)	Social Distance, *d* (m)	Ventilation Rate, $Q_{m}/N$ (m^3^/h∙per)	Duration, *t* (h)
“Z22” train cabin ^(TB10621-2009)^	85	25	3.3	0.99	20	40.5
Public transportation (long bus) ^(GB9673-1996)^	60	13.7	2.55	1.0791	25	1.5
Designated transportation (subway) ^(GB50157-2003)^	100	22	3	0.81	20	1

**Table 4:** The parameters used in the simulation for different roles of individuals.

Personnel Classification	Frequency of COVID-19 Tests ($f_{t}$)	Detection Accuracy (δ)	Initial Vaccina-tion Rate ($V_{\mathrm{initial}}$)	Vaccine Protec-tion Coefficient, ($v_{e}$)	Masking Rate, ($f_{I},f_{s}$)
Athletes	1 test/1 day	80%	85%	70%	90%
Olympic officials	1 test/2 days	80%	85%	70%	90%
Media	1 test/4 days	80%	85%	70%	90%
Local staff	1 test/2 days	80%	85%	70%	90%
Japanese residents	-	-	25%	70%	75%

**Table 5:** Parameter settings for SIQRV model in base case.

Parameters	Default Value	Reference
β	0.6~0.7	[42]
γ	0.1	[42]
$v_{e}$	0.7	[42]
ξ	1/60	[42]
$R_{0}$	6~7	[42]
$\Delta N_{v}$	78,176	[6]
N	13,960,000	[6]

## Data Availability

The data are publicly available at websites cited on references [6,11,14].
